# The removal of Zn from complex circumneutral pH mine waters using magnetic nanoparticles (MNPs)

**DOI:** 10.1039/d5en01049g

**Published:** 2026-01-09

**Authors:** Katie E. B. O'Neill, Jagannath Biswakarma, Rich Crane, James M. Byrne

**Affiliations:** a School of Earth Sciences, University of Bristol Bristol BS8 1TH UK katie.oneill@bristol.ac.uk james.byrne@bristol.ac.uk; b Camborne School of Mines, University of Exeter Penryn TR10 9EZ UK

## Abstract

Mine water discharges pose a significant environmental challenge due to elevated metal concentrations, which can be detrimental to aquatic ecosystems and water quality. In this study, four circumneutral-pH mine water samples were treated with different magnetic nanoparticle (MNP) concentrations (0.1 g L^−1^, 1 g L^−1^, and 5 g L^−1^) to assess their efficacy for Zn removal. Sorption of Zn to all MNP systems tested, occurred within 48 hours. At 5 g L^−1^, MNPs removed Zn from all mine waters tested, reducing concentrations to 0.09, 0.66, 0.0 and 0.0 mg L^−1^ for the River Ystwyth, Cwmystwyth adit, River Nent and Haggs adit respectively. A clear positive correlation was recorded for Zn removal as a function of MNP dose, with MNP concentrations >1 g L^−1^ required for Zn removal to below trace concentrations. Analysis of competing ions (*e.g.*, Ca^2+^, Mg^2+^, Na^+^) showed that a decrease in concentration followed the order Zn > Na^+^> Ca^2+^ > Mg^2+^. These findings confirm that MNPs are effective for the removal of Zn from real mine water samples even when applied at low dosages, suggesting that they are a highly promising water treatment technology for such applications.

## Abstract 2

Mine water discharges pose a significant environmental challenge due to elevated metal concentrations, which can be detrimental to aquatic ecosystems and water quality.

## Graphical abstract

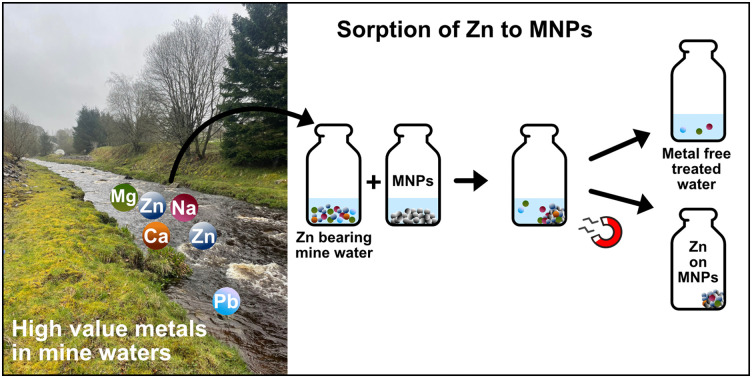

**Environmental significance**

In this study, we applied magnetic nanoparticles (MNPs) to remove zinc from circumneutral mine waters collected from two different locations in the United Kingdom, each characterised by distinct geochemical conditions and metal concentrations. The results show that magnetic nanoparticles can effectively remove zinc from mine waters, highlighting their potential for scalable and cost-effective water treatment solutions.

## 1. Introduction

Increasing demand for critical and strategically significant metals, driven in part by their vital role in renewable and clean energy technologies, is putting substantial pressure on global metal resources. Whilst metals are integral for modern industries, their release into the environment particularly through waste products from mining activities can lead to significant ecological degradation.^1,2^ As mining activities expand, the associated environmental damage will likely escalate. Resultingly, there is an urgent need for new approaches that, not only reduce the environmental impact of metal mining, but also recover valuable metals from mining waste products, that in turn decrease the demand for primary metal extraction.

Mine water contamination represents one of the most pressing environmental challenges worldwide.^2^ However, mine waters also contain valuable metal resources, and, unlike solid mine waste, offer a more consistent and continuous source of metals due to their ongoing formation.^3^ Circumneutral-pH mine waters, formed by the dissolution of carbonate minerals in metal-rich deposits, can contain high concentrations of important elements like zinc (Zn), lead (Pb), copper (Cu) and gold (Au). Although less studied than Acid Mine Drainage (AMD), these waters are widespread in occurrence and require effective remediation strategies.

Notable examples of circumneutral-pH mine waters include those from Zn–(Pb–Ag) mining operations, such as the Angouran mine in Iran, whereby, Zn concentrations reached 8 mg L^−1^ in an adit (pH 7.5) and 27 mg L^−1^ in a pit (pH 7.6).^4^ Similarly, historical mining in Leszczyna, Poland, has contributed between 120–330 μg L^−1^ of Zn in the Prusicki Potok stream (∼pH 7.4).^5^ Another example is in Mexico, whereby Zn concentrations of 1.73–67.60 mg L^−1^ and Pb concentrations of 1.92 mg L^−1^ have been recorded in circumneutral-pH waters associated with a nearby Zn–Pb–Cu mine.^6^ These examples indicate that circumneutral Zn- and Pb-bearing mine waters occur in diverse geological settings worldwide. The UK sites investigated in this study are consistent with these international systems in terms of pH, metal concentrations, and ionic composition, suggesting that the processes observed here may be broadly relevant to other circumneutral-pH mine waters. Metals like Zn are essential micronutrients, but, at elevated concentrations they can be toxic for the surrounding ecosystem, reducing biodiversity and compromising water quality.^7–10^ Addressing this contamination is critical for the sustainable management of water resources, particularly in regions where legacy pollution remains a concern.^11–14^ Insights gained from MNP performance in these UK waters can inform remediation strategies in comparable systems globally, particularly where Zn contamination poses environmental risks.

In the UK, circumneutral-pH mine waters are prevalent due to the historical mining of Zn, Pb and Cu bearing ores (along with other metals).^15^ The mining of these ores has been dated back to the Bronze Age, leading to many now abandoned metal mines across 13 main regions, including central Wales and the Northern Pennines.^15–17^ The region's geology is dominated by calcareous carboniferous rocks,^18^ which results in mine waters that are characteristically high in hardness and alkalinity with a neutral pH (pH 7.4–8),^19–21^ containing not only Zn but Cd and As.^16,19^

To mitigate circumneutral-pH mine water a range of active and passive treatment systems have been investigated.^22,23^ Existing active treatments, such as chemical precipitation and ion exchange require continuous input of chemicals and energy, making them expensive for long-term operation^23^ and sensitive to variations in water chemistry. Passive treatment, including wetlands, limestone drains, and anaerobic bioreactors, offer a more sustainable alternative but, still require maintenance and have limited efficiency under varying geochemical conditions such as changes in temperature, precipitation and redox conditions.^22,24–27^ Additionally, complex mine waters contain competing ions and elevated sulfate concentrations that can interfere with metal removal. These limitations highlight the need for a versatile, efficient and selective remediation strategy. Other alternative methods such as electrochemical or photocatalytic approaches, can remove multiple pollutants simultaneously, however, they often require specialised infrastructure, continuous energy input and careful control of operating conditions, which may limit their applicability in mine water treatment.

Adsorption based techniques have emerged as a promising alternative because of their flexibility, high removal efficiency and adaptability to varying water chemistries. They have been proven as effective for water purification across a wide range of industrial applications but remain largely unexplored for circumneutral-pH mine water treatment to date. Adsorption is relatively simple to implement, cost-effective, and is known to achieve high removal of target metal contaminants.^28,29^ However, a key challenge remains in achieving selective adsorption of target metals from complex matrices. Developing adsorbents with high selectivity for contaminants such as Zn or Pb should therefore, be a priority for circumneutral-pH mine water remediation.

Magnetic nanoparticles (MNPs) show considerable promise for mine water treatment due to their unique physicochemical properties^30,31^ including high surface area, tunable surface chemistry, and strong affinities for metal ions. In this study we use iron-oxide based MNPs (magnetite), which are low cost, abundant, and easily recoverable *via* magnetic separation, making them suitable for sustainable water treatment applications. Their tunable surface chemistry and the capacity for surface functionalisation enables targeted interactions between metal ions and the nanoparticle surface.^32–37^ Surface functionalisation of adsorbents using ligands such as carboxyl (–COOH) have the potential to enhance metal removal by increasing the number of specific metal binding sites and therefore, strengthening possible inner sphere complexation. For example, surface modification of biochar demonstrated improved uptake of naphthenic acids compared to non-activated biochar.^38^ However, in the present study unmodified iron-oxide MNPs were deliberately employed to establish baseline Zn removal performance under environmentally realistic conditions prior to further material optimisation.

In addition to this, their (super)paramagnetic properties allow for rapid and efficient separation from treated water using external magnetic fields^39^ simplifying recovery and reducing the need for post-treatment processing. Their magnetic properties also allow MNPs to be recovered and reused, maintaining high Zn removal efficiency over multiple treatment cycles. Furthermore, engineered hybrid composites of MNPs and supporting materials have demonstrated enhanced adsorption capacity and recyclability for cationic pollutants^38^ suggesting future avenues to further improve Zn removal efficiency. This combination of reactivity, selectivity and recoverability contributes to their potential as a possible cost-effective and efficient option for large-scale applications.^31,40^

Despite growing interest in MNPs, research on their application to treat circumneutral-pH mine water remains limited. Existing studies have often focussed on examining metal removal within synthetic chemical solutions. This does not fully capture the complexity of real world mine waters.^41,42^ Factors that can impact adsorption efficiency include pH, redox conditions, and co-contaminants, which are often overlooked in simplified lab studies. This knowledge gap highlights the need to evaluate MNP performance under environmentally relevant conditions. In particular, studies on Zn removal using MNPs in circumneutral-pH mine waters are extremely limited. By addressing this, the present study provides novel insights into the practical application of MNPs, demonstrating their potential for targeted metal removal.

This research investigates the use of MNPs to remove Zn from circumneutral-pH mine waters from two UK locations with varying geochemical conditions and metal concentrations. The objectives are: i) to determine the effectiveness of using MNPs in reducing Zn concentrations to below the environmental quality standards (EQS) in environmental samples; ii) to investigate whether Zn is removed by MNPs in the presence of background competing ions; iii) to elucidate the best MNP concentration for optimum Zn removal. This work aims to contribute to closing the gap between laboratory research and real-world environmental applications. This is by advancing the development of sustainable adsorbents for circumneutral-pH mine water metal remediation, whilst also moving towards a circular economic approach.

## 2. Methodology

### 2.1 MNPs synthesis

MNPs were synthesised and characterised as shown in the previous study conducted by O'Neill *et al.*, (2025).^43^ The same batch of MNPs from that study was used herein.

### 2.2 Mine water sites and collection

**Fig. 1:**
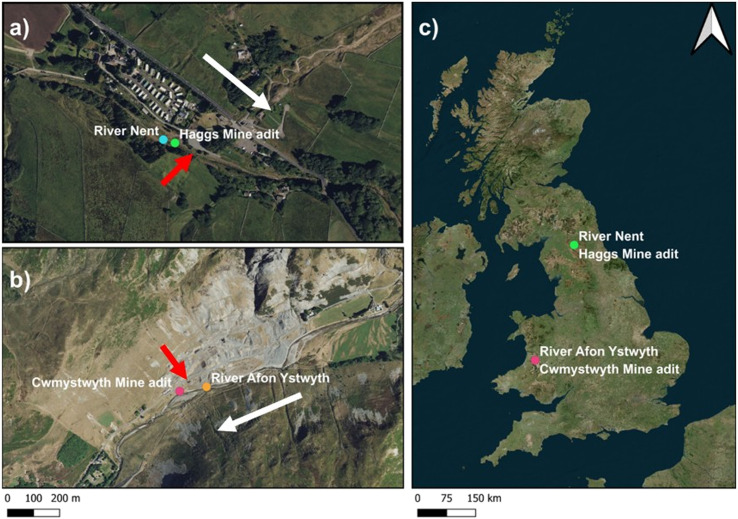
A map of the sample locations with a) the North Pennines location with the River Nent shown as a blue circle and Haggs adit shown as a green circle and b) the Ystwyth valley location with Cwmystwyth adit shown as a pink circle and the River Ystwyth shown as an orange circle. c) Depicts the locations in context of the United Kingdom. White arrows indicate the direction of water flow for the rivers, and red arrows indicate the direction of flow of water for the adits. Map created using QGIS.

Water samples were collected from four locations (Fig. 1): two along the River Nent (England) and two along the River Ystwyth (Wales). At each site, one sample was taken from a mine adit and the other was collected directly from the river which are fed by both mine adits and run-off from leached mine waste.

Samples were collected in 10 L polyethylene containers that were pre-rinsed with nitric acid (HNO_3_), tap water and site water before filling. They were filtered under vacuum filtration using 0.45 μm filter paper and stored at 4 °C prior to use.

*In situ* geochemical properties were recorded at each site, including pH, temperature and conductivity using a HACH HGd portable meter.

### 2.3 Study site 1: Nent Valley

The River Nent is located in northeast Cumbria, within the Nent Valley which forms part of the Alston Block. This is the most northern section of the Northern Pennine Orefield.^18^ The Alston Block contains extensive Pb and Zn ore deposits leading to signifcant galena (PbS) and sphalerite (ZnS) mining.^24^ Mining operations persisted for over two centuries, resulting in the construction of over 90 adits within the valley.^21^ Several of these adits continue to discharge into the River Nent, contributing elevated concentrations of metals such as Pb and Zn.^22^ Samples were collected from the River Nent, a tributary of the South Tyne that is within the Tyne catchment (British National Grid (BNG): 376470, 544998) and Haggs adit (BNG: 376491, 544992).

### 2.4 Study site 2: Ystwyth Valley

The Ystwyth valley is southeast of Aberystwyth and is a part of the ore fields of north Cardiganshire.^17^ Mining in this region dates back to the early Bronze Age, primarily targeting Zn, Pb, Cu and Au.^17^ Numerous abandoned mines with exposed waste and mine water discharges remain, many of which fail to meet UK water quality targets.^13^ The River Ystwyth is heavily impacted by past mining activities and receives both surface and sub-surface drainage from the Cwmystwyth mine (52°21′23″N, 003°45′38″W).^11^ This mine covers approximately 250 ha on the northern slopes of the Ystwyth valley^12,13^ and is estimated to release 12 tonnes of Zn and 2 tonnes of Pb into the river annually.^12,13^ The remains of historical mining is still visible in the surrounding landscape with waste dumps, buildings, shafts and adits.^11^ Samples were taken directly from Cwmystwyth adit (British National Grid (BNG): 280094, 274400) and upstream from in the River Ystwyth (BNG: 280094, 274400) both within the Teifi catchment.

### 2.5 Sorption experiments

Adsorption experiments were performed to understand the interaction between MNPs and the metals present within an environmental sample over 48 hours. All experiments were performed in triplicate with a total volume of 150 mL of water sample at ambient temperature. Filtered water samples were degassed with N_2_ before being added into the reactor bottles. Three concentrations of MNPs were tested and included: 0.1 g L^−1^, 1 g L^−1^ and 5 g L^−1^. Before MNPs were added to the reactors the pH was measured, then once added, the pH was measured again. The final pH was also recorded at 48 hours. Samples for analysis were collected before the addition of MNPs and taken at time intervals 0, 0.033, 0.0667, 0.17, 0.5, 1, 1.5, 2, 4, 6, 24, 48 hours. Samples were centrifuged at 5000 rpm for 5 minutes. The supernatants were collected for elemental analysis.

The metal concentration of each supernatant (*C*_e_) was measured using an Agilent 5110 ICP-OES under the following operating conditions: a plasma argon flow rate at 12 L min^−1^, auxiliary flow rate at 1 L min^−1^, read time at 5 seconds, nebulizer argon flow rate at 0.7 L min^−1^. The instrument was operated in axial viewing mode. Calibration curves for each element were generated using standard stock solutions and each element exhibited good linearity with *R*^2^ values around 0.99. The detection limit for Zn in water was 0.9 ng L^−1^, which is below the environmental quality standards (EQS) for UK surface waters (Tyne catchment: 0.0157 mg L^−1^; Teifi catchment: 0.0134 mg L^−1^).

Ion chromatography (IC) separation was conducted using a Thermofisher Dionex ICS 5000. Cation concentrations for magnesium, calcium, sodium and potassium were determined by injecting 10 μL of sample into a CS12A (2 × 250 mm) column with a CG12A (2 × 50 mm) column guard at 35 °C. The eluent used was methanesulfonic acid at 0.4 mL per minute at gradient concentration. The same ion chromatogram was used for anions: fluoride, chloride, nitrate, sulphate and phosphate. The column used was AC11-HC (2 × 250 mm) with column guard Ag11-HC (2 × 50 mm) at 30 °C with sample injection volume of 10 μL. The eluent for anions was potassium hydroxide (isocratic at 23 mM) at 0.38 mL per minute.

The Langmuir equation was used to describe the combined collected data with a previous data set and to determine the maximum adsorption capacity.1.1
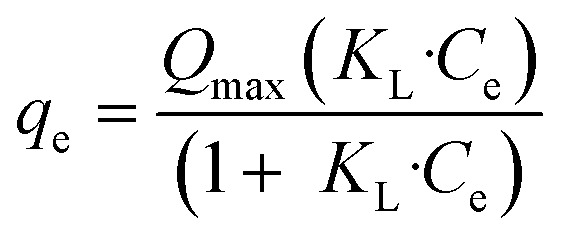
where:

*q*_e_ is the amount of adsorbate adsorbed per unit mass of adsorbent (mg g^−1^),

*Q*_max_ is the maximum adsorption capacity (mg g^−1^),

*C*_e_ is the final equilibrium concentration of Zn and ions in the solution (mg L^−1^) after treatment,

*K*_L_ is the Langmuir adsorption coefficient.

To simplify eqn (1.1), we used the following linearised form of the equation:1.2
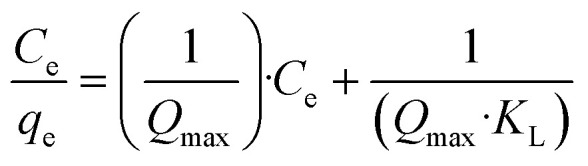
With a linear regression line fit to the data, the slope and the intercept were used to determine *Q*_max_ and *K*_L_. Based upon these obtained values the parameters were optimised to best fit the data in non-linearised form. Excel solver function was used to optimise these parameters.

## 3. Results and discussion

### 3.1 Water sample composition

**Fig. 2:**
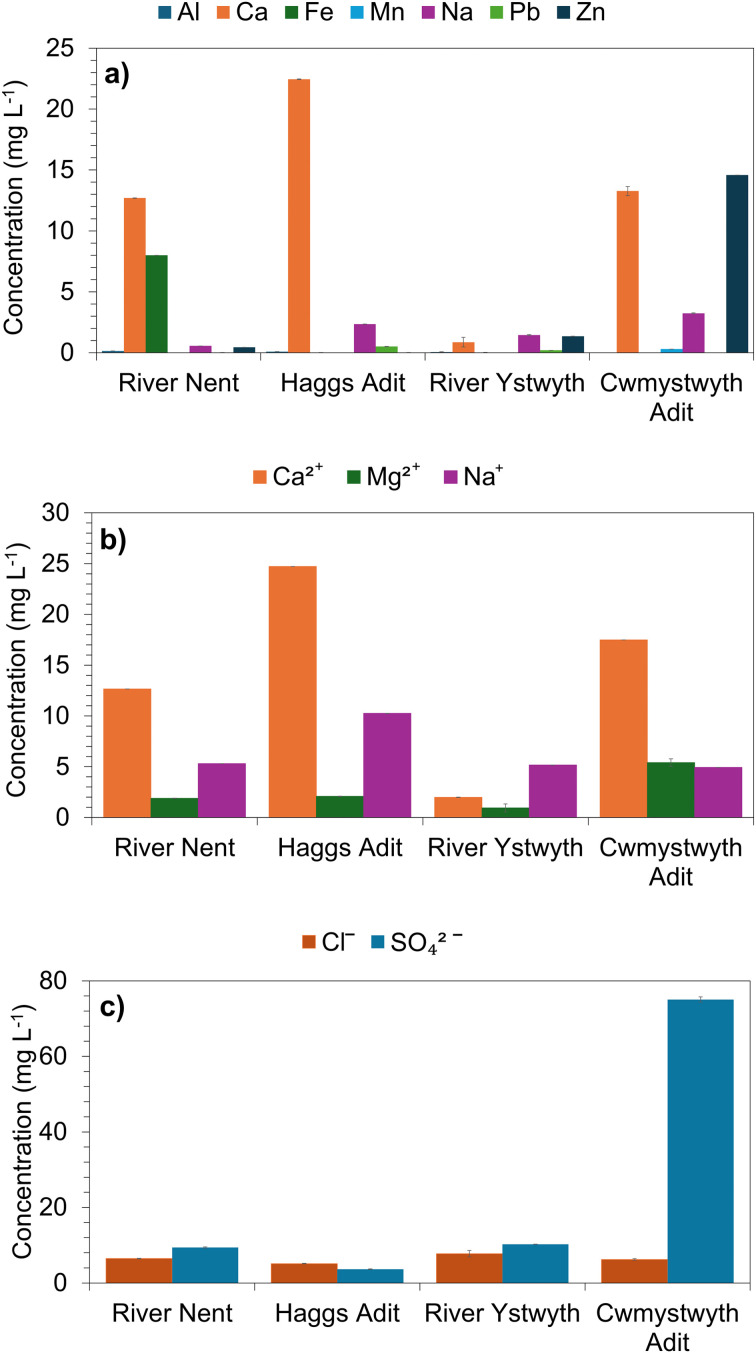
The concentrations of elements, cations, and anions found in field samples collected from four locations: River Nent, Haggs adit, River Ystwyth, and Cwmystwyth adit. Panel a) illustrates the concentration (mg L^−1^) of Al, Ca, Fe, Mn, Na, Pb, and Zn, determined through ICP-OES. The concentrations of b) cations and c) anions (mg L^−1^), measured *via* IC, are also presented for these sites. Error bars denote standard deviations from triplicate measurements.

Mine water samples from the four locations were characterised for their dissolved elemental composition using ICP-OES. As shown in Fig. 2a, for the River Nent sample Ca (12.7 mg L^−1^) and Fe (8 mg L^−1^) are the most dominant elements with lower concentrations of Al, Mn, Na, Pb and Zn (less than 0.58 mg L^−1^). In contrast there was no Fe detected in the water sample collected from the Haggs adit; however, the Ca concentration (22.45 mg L^−1^) was almost two times higher than the River Nent, making it the highest Ca concentration across the sites. Haggs adit also contained more Pb (0.52 mg L^−1^) than the River Ystwyth (0.2 mg L^−1^). However, the Zn concentration was highest in the Cwmystwyth adit (14. 58 mg L^−1^) followed by Ca (13.27 mg L^−1^) and Na (3.24 mg L^−1^).

Cation (Ca^2+^, Mg^2+^ and Na^+^) concentrations measured using IC are shown in Fig. 2b. For the River Nent and Haggs adit samples, there is a consistent trend with the concentration from highest to lowest following the order of Ca^2+^ > Na^+^ > Mg^2+^. This is not the case within the River Ystwyth and Cwmystwyth adit. Generally, in the River Ystwyth there were lower concentrations of cations compared to the River Nent and Haggs adit. Whereas, Cwmystwyth adit had the highest concentration of Mg^2+^ of all the sites with similar concentrations of Ca^2+^ and Na^+^.

Anions, measured using IC (Fig. 2c), showed much higher concentrations of SO_4_^2−^ (75 mg L^−1^) in the Cwmystwyth adit than at the other sites (River Nent: 9.39 mg L^−1^, Haggs adit: 3.66 mg L^−1^, River Ystwyth: 10.27 mg L^−1^). There were however, similar concentrations of Cl^−^ in all samples.

The variations in elemental concentrations across the four sites can be attributed to differences in the underlying geology, historical mining activities and hydrological conditions in the two geographical catchments.^44^ The geology of Penrith in England (the area covering the River Nent and Haggs adit) as stated by Dean *et al.*^45^ is predominantly bioclastic limestone, sandstone, mudstone, siltstones and rare coals. In contrast, the Ystwyth valley in Wales is comprised mostly of lower Silurian sandstones, siltstones and mudstones.^46^ Given the dominant geology in Penrith is limestone, this could account for the higher concentrations of Ca present at these sites.^44,47^

**Fig. 3:**
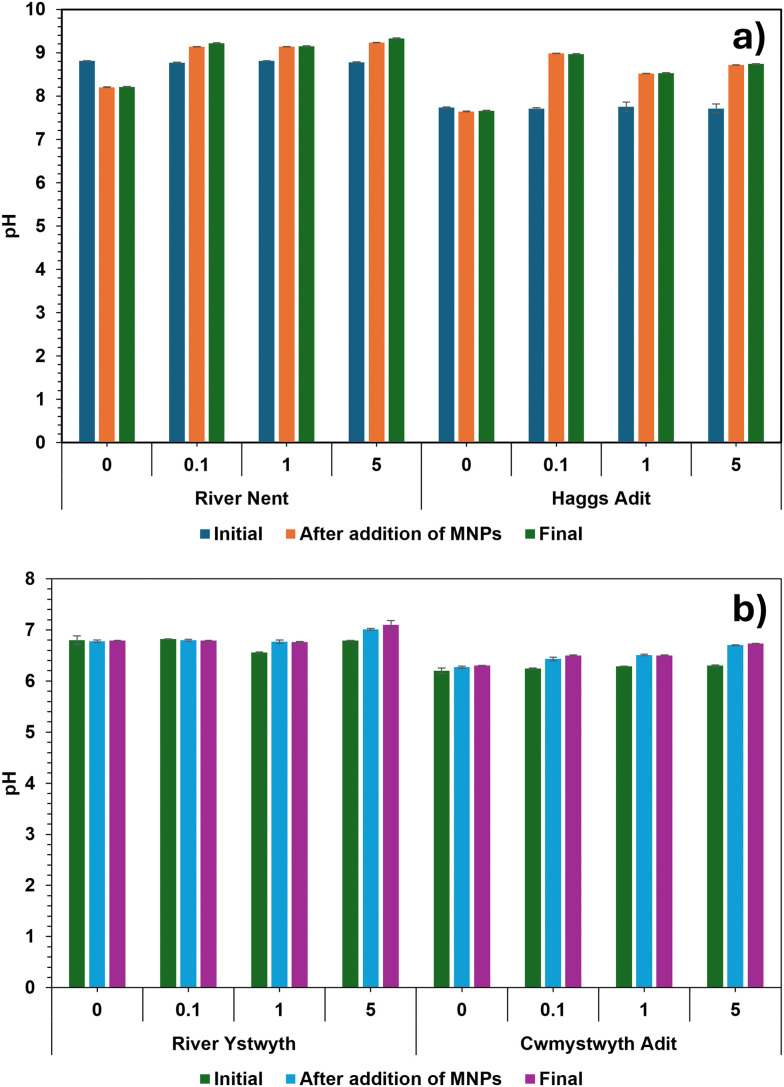
Effect of 0, 0.1, 1, 5 g L^−1^ of MNPs, on mine waters from River Nent, Haggs adit, River Ystwyth and Cwmystwyth adit. Fig. 3a shows the pH before MNPs (dark blue), upon addition of MNPs (orange) and after 48 hours (green) for the River Nent and Haggs adit. Whereas, Fig. 3b, shows the pH before MNPs (green), upon addition of MNPs (light blue) and after 48 hours (purple) for the River Ystwyth and Cwmystwyth adit. Error bars represent the standard deviation of triplicate measurements.

As per the USGS (United States Geological Survey) definition of water hardness,^48^ the following equation was used to examine the water hardness of the four sites.Total water hardness (mg L^−1^) = [Ca] (mg L^−1^) × 2.497 + [Mg] (mg L^−1^) × 4.118The hardness of the samples from the River Nent and the River Ystwyth were soft (<60 mg L^−1^) whereas, Haggs adit and Cwmystwyth adit were moderately hard (61–120 mg L^−1^). This aligns with their recorded pH values of ∼8.8 for the River Nent, ∼7.5 for Hagg's adit, ∼6.8 for the River Ystwyth and ∼6.2 for Cwmystwyth adit (Fig. 3). Notably, in Fig. 3, the pH changes between the field and once back in the laboratory. This is due to the degassing of CO_2_ from the water samples because dissolved CO_2_ in the water forms carbonic acid, and when CO_2_ degases this reduces the acidity and thus increases the pH, similarly to what is observed in this study.

In addition to Ca, Fe can also be an indicator of the underlying geology. The Fe concentrations were generally low across the sites, except for the River Nent (8 mg L^−1^). The presence of Fe here may be due to the weathering of iron-bearing minerals such as the Iron Post limestone.^49,50^ This is a marine horizon located over the Alston Block (Pennines), which is mostly unexposed; however, in Nenthead, where the River Nent flows, there is a section exposed at the surface.^49,50^ This section is therefore vulnerable to weathering and dissolution that in turn, may increase the concentration of Fe in the River Nent.^49,50^ The low Fe in the other sites suggests an absence of Fe minerals or that any Fe present is precipitated as Fe oxyhydroxides or amorphous Fe, which would remove Fe from the aqueous phase.

The occurrence of Zn and Pb, particularly at the mine adits, is attributed to the presence of Zn–Pb deposits in the form of galena (PbS) and sphalerite (ZnS), which were extensively mined at both geographical locations.^11,17,18,20,51^ The legacy of mining these ores has resulted in widespread land and water contamination.^20^ Metals like Zn and Pb as well as sulphate anions (SO_4_^2−^) are leached from spoil heaps and tailings into the surrounding environment predominantly through rivers and mine adits.^19^

Our results, in Fig. 2a and c, show that the highest concentrations of Zn and SO_4_^2−^ were found in samples from the Cwmystwyth adit (Zn: 14.58 mg L^−1^, SO_4_^2−^: 75 mg L^−1^). The elevated concentration of SO_4_^2−^ is likely due to a higher abundance of sulphide minerals and/ or increased surface exposure of sulphide-bearing rocks to water and oxygen. Notably, these samples also had lower pH values compared to other sites (see Fig. 3), which could accelerate sulphide oxidation, resulting in higher SO_4_^2−^ concentration being released. The occurrence of both Zn and SO_4_^2−^ supports the proposal of possible chronic leaching of sulphide minerals at this site.

In contrast, Zn mobility was more limited at the other locations highlighting the importance of site specific environmental controls like pH, redox conditions, and mineralogy for determining metal transport. Similarly to Zn, Pb concentrations are also influenced by these geochemical factors,^52^ particularly pH and alkalinity. Although Pb bearing ores are present at both geographical locations, Pb was only detected at two sites: Haggs adit (0.52 mg L^−1^) and the River Ystwyth (0.2 mg L). This further supports that metal mobility and occurrence are strongly influenced by specific geochemical conditions.

Another environmental parameter that influences metal mobility is ionic strength. Na^+^ and Cl^−^ present in each site serve as electrolytes to further control mineral precipitation and therefore dissolution of heavy metals.^53^ A 1 : 1 ratio of Na^+^ and Cl^−^ were used to explain the buffering capacity of those systems. From the data collected, the concentration of NaCl can be determined and was found to be similar across the sites with some slight variations (River Nent: 0.09 mMol L^−1^, Haggs adit: 0.18 mMol L^−1^, River Ystwyth: 0.09 mMol L^−1^, Cwmystwyth adit: 0.08 mMol L^−1^).

In the context of water quality regulations, the measured Zn and Pb concentrations within all the water samples tested exceed the current environmental water quality standards set by the UK Technical Advisory Group.^54^ The Water Framework Directive (WFD) reports the environmental quality standards (EQS) for priority substances in surface waters. As Zn is naturally occurring in high concentrations, the EQS is set for individual catchments with reference to local water chemistry and background concentrations.^54^ Taking these into account, the maximum allowable concentrations of Zn should not exceed 0.0157 mg L^−1^ in freshwaters in the Tyne catchment, or 0.0134 mg L^−1^ in freshwaters for the Teifi catchment. All sites were observed to exceed this limit, particularly Cwmystwyth adit (14.58 mg L^−1^), which exceeded the threshold by 1000 fold.

Similarly, maximum allowable concentration for Pb is 0.0013 mg L^−1^ for inland surface waters^54^ and is not catchment dependent. Where Pb was detected, it exceeds this threshold (Haggs adit: 0.52 mg L^−1^, River Ystwyth: 0.2 mg L^−1^). Therefore, both Zn and Pb do not meet the EQS and indicate serious concerns for the health of the surrounding environment, specifically on the aquatic ecosystem which is known to be extremely vulnerable at such concentrations. As a result, it is important to remove these metals from such mine waters.

### 3.2 Zn removal by MNPs across different mine waters

**Fig. 4:**
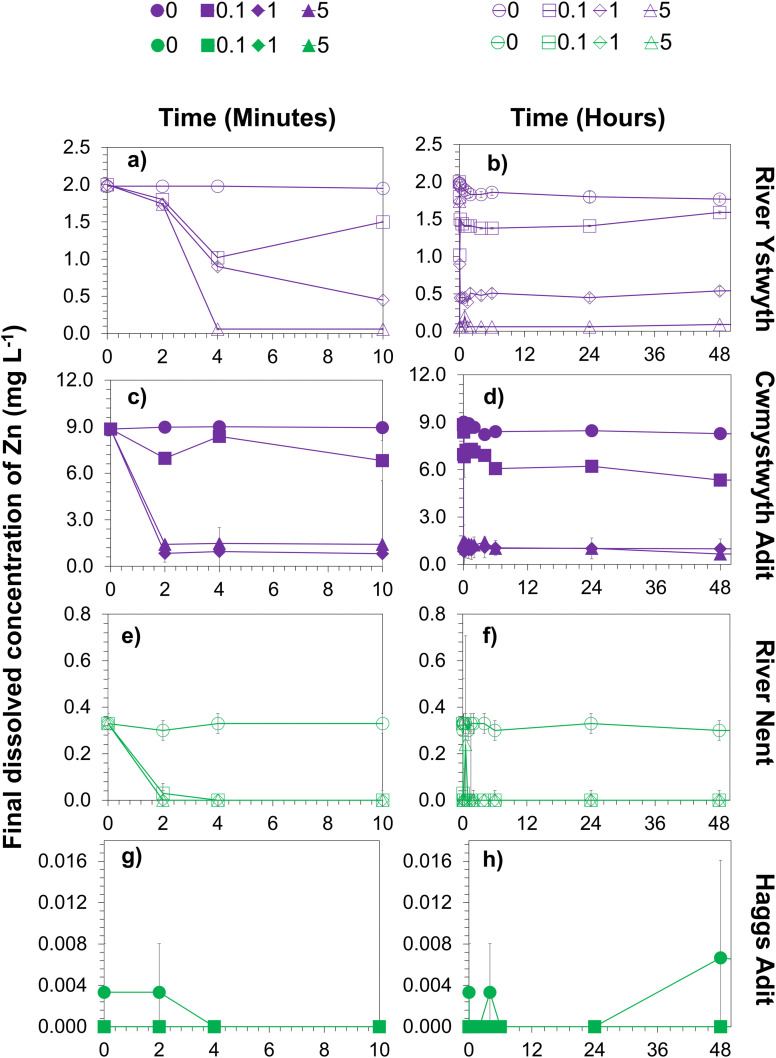
Final dissolved Zn concentrations in mine water samples from the River Ystwyth (a and b), Cwmystwyth adit (c and d), the River Nent (e and f), and Haggs adit (g and h), after exposure to varying magnetite nanoparticle (MNP) concentrations (0, 0.1, 1, and 5 g L^−1^) under anoxic conditions for 48 h. Panels on the left (a, c, e and g) show Zn concentrations during the first 10 min of the experiments, while panels on the right (b, d, f and h) show the full 48 h duration. Open symbols represent rivers and closed symbols represent mine adits. Line and symbol colours correspond to site locations: green for the River Nent and Haggs adit, and purple for the River Ystwyth and Cwmystwyth adit. Symbols indicate MNP concentrations: circles (0 g L^−1^), squares (0.1 g L^−1^), diamonds (1 g L^−1^), and triangles (5 g L^−1^). Error bars represent standard deviations from triplicate measurements.

Sorption experiments were conducted using varying concentrations of MNPs added to the mine water samples. Changes in final dissolved Zn concentrations over time are shown in Fig. 4. The initial Zn concentrations were highest in the Cwmystwyth adit (8.9 mg L^−1^), followed by the River Ystwyth (2.0 mg L^−1^), the River Nent (0.3 mg L^−1^) and Haggs adit (0.004 mg L^−1^) as shown in Table 1. For all mine water samples tested, the most substantial decrease in dissolved Zn occurred within the first 4 minutes (Fig. 4a, c, e and g), after which Zn concentrations remained stable over the 48-hour period, indicating an absence of Zn desorption. The rapid removal of Zn highlights the importance of effective contact between MNPs and dissolved metals. Efficient interaction is a key factor controlling treatment effectiveness, specifically in complex aqueous systems. This is consistent with previous studies showing that optimizing reagent delivery and transport can substantially enhance contaminant removal.^55^

**Table 1:** Initial Zn concentration, Environmental Quality Standards (EQS) and pH for each site

Site	Sample type	Initial Zn (mg L^−1^)	EQS (mg L^−1^)	pH
River Nent	River	0.30	0.0157	8.8
Haggs adit	Adit	0.004	0.0157	7.5
River Ystwyth	River	2.00	0.0134	6.8
Cwmystwyth adit	Adit	8.90	0.0134	6.2

For the mine water samples collected from Cwmystwyth adit and River Ystwyth, which contained the highest concentrations of Zn, Zn removal increased with increasing MNP concentration (Fig. 4b and d). In contrast, for the River Nent and Haggs adit samples, which had lower initial Zn concentrations, complete removal was achieved across all MNP concentrations (Fig. 4f and h). These observations are consistent with Langmuir-type behavious whereby, removal efficiency depends on the ratio of solute to available surface sites on the adsorbent. In contrast, Zn was removed onto MNPs to below detection limit for the samples taken from the River Nent and Haggs adit (Fig. 4f and h).

For samples with higher initial Zn (Cwmystwyth adit: 8.9 mg L^−1^, River Ystwyth: 2.0 mg L^−1^), a clear MNP concentration dependence was observed. Higher MNP concentrations led to higher Zn removal. This suggests that sorption capacity increased with the number of available surface sites. In the River Nent and Haggs adit (Fig. 4e–h), lower initial Zn concentrations facilitated fast and complete removal across all MNPs concentrations.

It should be noted, that the initial Zn concentrations in samples from the River Nent (0.30 mg L^−1^) and Haggs adit (0.004 mg L^−1^) were considerably lower than those at Cwmystwyth adit and the River Ystwyth. In particular, samples from Haggs adit contained concentrations closer to the detection limit of ICP-OES (0.00009 mg L^−1^). Therefore, the complete removal of Zn in these sites primarily reflects the low starting concentrations. Conversely, for high-Zn samples, MNP concentration and contact time had a more pronounced effect on removal efficiency, highlighting the importance of optimising treatment conditions for sites with elevated contamination.

When combining data from this study and that by O'Neill, *et al.* (2025)^43^ the Langmuir isotherm best described the dataset (Fig. S1 and S2) with a calculated maximum capacity of *Q*_max_ = 26.80 mg g^−1^. This *Q*_max_ was compared to other adsorbents in Table 2, whereby modified adsorbents generally displayed a higher sorption capacity. However, as the data sets were not collected under comparable conditions it is difficult to determine the applicability and reliability of this calculated *Q*_max_.

**Table 2:** Zinc sorption experiments: Maximum sorption capacity (*Q*_max_ mg g^−1^) calculated using Langmuir equation

Adsorbent	Adsorbent concentration (g L^−1^ unless stated)	pH	*Q*_max_ a	*K*_L_	*R* ^2^	Reference
MNPs	5	7	26.80	0.02	0.88	This study
Sodium dodecyl sulphate-coated Fe_3_O_4_	15 mg Fe_3_O_4_ +25 mg SDS	6	56.20	0.26	0.99	56
Magnetite/carbon nanocomposite	1	6.1	42.90	0.03	0.99	57
Bentonite coated with synthesized Fe_3_O_4_	8	6	22.60	0.10	0.99	58
Graphine oxide	0.5	6	121.00	0.11	0.95	59
Magnetite	0.5	6	7.70	1.22	0.84	59
Graphine oxide : magnetite (2 : 1)	0.5	6	48.40	0.25	0.93	59
Graphine oxide : magnetite (1 : 1)	0.5	6	22.00	2.22	0.98	59
Graphine oxide : magnetite (1 : 2)	0.5	6	19.60	0.34	0.96	59
Magnetite	4	6	52.60	0.02	0.95	60
Baobab	4	6	24.40	0.15	0.99	60
Magnetite–baobab composite	4	6	37.00	0.19	0.92	60
Purified commercial single-walled carbon nanotubes	0.5	7	43.66	0.19	0.99	61
Purified multiwalled carbon nanotubes	0.5	7	32.68	0.22	0.99	61
Powdered activated carbon	0.5	7	13.40	0.07	0.99	61

a *Q*_max_ was calculated using the simplified linearised Langmuir eqn (1.2).

### 3.3 Influence of background ions on Zn sorption

**Fig. 5:**
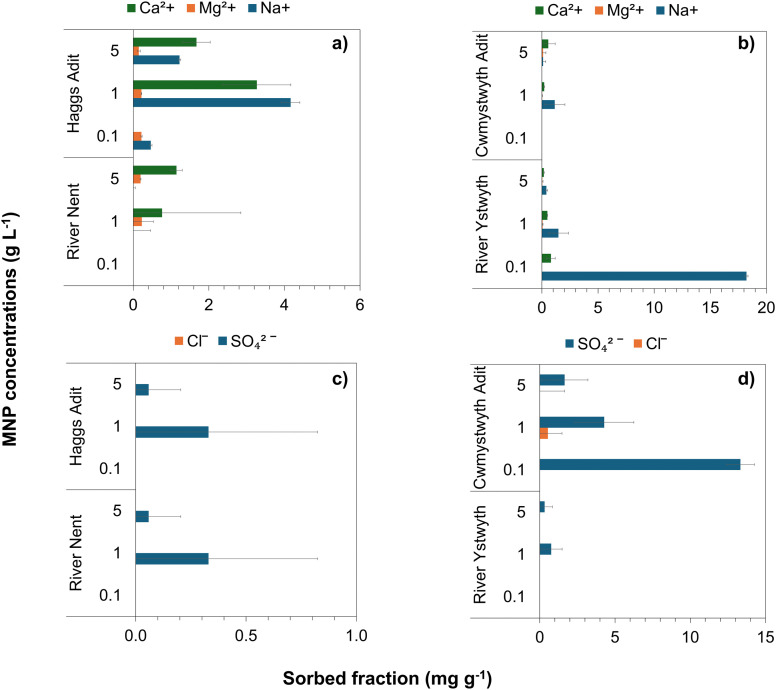
Sorbed fractions (*q*_e_) of cations (Ca^2+^, Mg^2+^, Na^+^) and anions (SO_4_^2−^, Cl^−^) onto magnetite nanoparticles (MNPs) from four circumneutral mine water samples at varying MNP concentrations (0.1, 1, and 5 g L^−1^) under anoxic conditions. (a) Cation sorption from Haggs adit and the River Nent, (b) cation sorption from Cwmystwyth adit and the River Ystwyth, (c) anion sorption from Haggs adit and the River Nent, and (d) anion sorption from Cwmystwyth adit and the River Ystwyth. Cation and anion colours are Ca^2+^ (green), Mg^2+^ (orange), Na^+^ (blue), SO_4_^2−^ (patterned blue), and Cl^−^ (patterned orange). Error bars represent standard deviations from triplicate measurements.

The sorption of non-target cations (Ca^2+^, Mg^2+^, Na^+^) and anions (SO_4_^2−^, Cl^−^) across different MNP concentrations was assessed using ion chromatography (Fig. 5). Some removal of competing ions was observed, particularly Ca^2+^ and Na^+^ in the River Nent and Haggs adit. For example, Ca^2+^ decreased from 25.2 to 17.0 mg L^−1^ in Haggs adit and from 12.6 to 6.3 mg L^−1^ in the River Nent. The extent of this removal however, was low relative to Zn. Similarly, only a small decrease in SO_4_^2−^ was seen in the Cwmystwyth adit from 75 to 69 mg L^−1^ at 5 g L^−1^ of MNPs. These results highlight the high selectivity of the MNPs for Zn, even in the presence of elevated background concentrations of other ions. Notably, in all mine water samples Zn was consistently reduced to near or below detection limits, whereas the concentrations of major ions remained largely unaffected. This demonstrated that MNPs can effectively target Zn in real, geochemically complex waters, which is a key advantage for practical environmental applications.

In the River Nent and Haggs adit, higher cation sorption is likely due to the increased availability of sorption sites due to lower initial Zn concentrations. In contrast, the Cwmystwyth adit exhibited the lowest cation sorption, likely because Zn outcompeted other background cations (Fig. S3). The uptake of Zn could be because of its high surface charge density which means it can form strong inner-sphere complexes with surface hydroxyl groups on the surface of the MNPs. In addition to this, more Na^+^ was sorbed compared to Ca^2+^ and Mg^2+^. This trend could be due to their differences in hydration enthalpy. Na^+^ has a lower charge density compared to Ca^2+^ and Mg^2+^ which means it has a weaker hydration enthalpy. As Na^+^ has a weaker hydration enthalpy it has therefore, a more loosely bound hydration shell. As a result, it can partially dehydrate eaiser and interact more readily with surface sites on the MNPs. On the other hand, Ca^2+^ and Mg^2+^ have stronger hydration enthalpies which limit their ability to dehyrate and form inner-sphere complexes with the MNP surface.

Additionally, at this site there was also the highest SO_4_^2−^ sorption measured, which could be a result of co-sorption (Fig. S4). Localised positively charged regions on the MNPs surface may be formed by Zn^2+^ which in turn promotes the electrostatic attraction of SO_4_^2−^ ions. The sorption of SO_4_^2−^ ions may reduce the number of available surface sites causing both Zn and SO_4_^2−^ to compete for the same sites. Additionally, the competition for surface sites, and changes to the MNP surface characteristics, could be influenced by other ions present in the solution, with other cations and anions potentially modifying surface interactions and affecting the overall efficiency of Zn removal.

Overall, the sorbed fractions of background ions were relatively and consistently low compared to Zn, despite being relatively high in aqueous concentrations. This indicates that MNPs have a strong preference for Zn, and suggests that Zn possibly forms more stable inner sphere surface complexes, whereas other ions may remain in solution or loosely attached to the MNP surfaces due to weaker outer sphere interactions. Previous research has shown that Zn commonly forms stable inner-sphere complexes with iron oxyhydroxide surfaces.^62–65^ While Zn was consistently removed by the MNPs, no significant Pb sorption was observed (Fig. S5). Preliminary control experiments assessing Pb sorption to experimental glassware were found to be inconclusive, with no consistent trend across replicates. This suggests that both container interactions and matrix effects could be contributing to the observed variability. Future Pb specific studies should employ isotope sensitive analyses and use surface passivated reactors to resolve Pb sorption behaviour in complex mine waters. As a result, Pb soprtion was not interpreted quantitatively in this study.

It should also be noted that environmental factors such as seasonal temperature fluctuations, can influence adsorption behaviour and metal uptake in natural waters. Previous studies have shown that freeze–thaw processes can regulate contaminant adsorption in soils,^66^ which highlights that MNP performance may vary under field conditions. However, MNPs are unlikely to be deployed directly in field conditions where seasonal variability occurs. Nonetheless, acknowledging that environmental factors can influence adsorption, highlights that MNP performance may vary in natural systems compared to controlled laboratory experiments.

## 4. Conclusions

The real-world water samples used in this study are all typical of Zn-bearing circumneutral-pH mine waters, but we have tested a range of elemental compositions within this that are representative of variability found in mine impacted river systems. Despite this, MNPs demonstrated selective removal of Zn consistently across the samples, this is a trait that is advantageous for water treatment technologies. From this, it could be said that MNPs would have a broad applicability for the treatment of a wide range of circumneutral pH Zn-bearing mine waters.

Currently, relatively few water treatment systems offer metal-specific selectivity or where such technologies exist, they can often be expensive and/or difficult to apply.^67,68^ For example, commonly, selective membranes are used in conventional water treatment. However, their efficacy is subject to fouling (permeability losses), and often they require significant energy input resulting in high operational costs and therefore, limiting scalability. On the other hand, MNPs are highly effective for Zn selectivity and do not require any pH adjustment or additional reagents. This coupled with their magnetic properties, they have the potential to be applied to open-flow systems, with their suspension and capture controlled by an external magnetic field.

Zn was removed in all samples to varying degrees, and importantly, in two samples with low initial concentrations (River Nent and Haggs adit), Zn was removed to below the detection limit and below the Environmental Quality Standard (EQS) for the Tyne catchment. However, in samples with higher initial Zn concentrations (River Ystwyth and Cwmystwyth Adit), Zn removal did not reach the EQS for the Teifi catchment. The findings support the potential of MNPs for targeted remediation of Zn contaminated waters but, performance optimisation such as modifying the MNP surface with additional functional groups is needed in order achieve maximum adsorption. Future optimisation may involve surface modification of MNPs with functional groups such as carboxyl groups to further enhance Zn selectivity and adsorption capacity in mine waters, building on the baseline performance demonstrated here.

The use of real mine waters revealed complexities absent in synthetic solutions, including competing ions, variable pH, and high sulfate concentrations. These challenges underscore the novelty of this study in demonstrating selective Zn removal by MNPs under realistic environmental conditions. Overall, MNPs offer a promising, cost-effective, and selective treatment method for Zn removal, which negates the need for chemical modification or energy-intensive treatment. This could bridge the gap in current remediation technologies, specifically for passive or decentralized water treatment in legacy mining regions. With further research and optimization, MNP-based treatment could offer scalable solutions that meet catchment-specific EQS targets while maintaining low operational demands.

## Author contributions

K. E. B. O.: conceptualization, methodology, investigation, writing – original draft, review & editing. J. B.: conceptualization, methodology, supervision, investigation, writing – review & editing. R. C.: conceptualization, supervision, writing – review & editing, funding acquisition. J. M. B.: conceptualization, supervision, writing – review & editing, funding acquisition, project administration.

## Conflicts of interest

The authors declare no competing financial interest.

## Supplementary Material

EN-013-D5EN01049G-s001

## Data Availability

The data supporting this article have been included as part of the supplementary information (SI). Supplementary information: SI presents additional Zn sorption isotherm data, along with final dissolved concentrations of major cations, anions, and Pb in mine water samples after treatment with magnetite nanoparticles. See DOI: https://doi.org/10.1039/d5en01049g.

## References

[cit1] Cappuyns V., Alian V., Vassilieva E., Swennen R. (2014). pH Dependent Leaching Behavior of Zn, Cd, Pb, Cu and As from Mining Wastes and Slags: Kinetics and Mineralogical Control. Waste Biomass Valoriz..

[cit2] Paktunc A. D. , Characterization of Mine Wastes for Prediction of Acid Mine Drainage, in Environmental Impacts of Mining Activities: Emphasis on Mitigation and Remedial Measures, ed. J. M. Azcue, Springer Berlin Heidelberg, 1999, pp. 19–40

[cit3] Calugaru I. L., Neculita C. M., Genty T., Zagury G. J. (2018). Metals and metalloids treatment in contaminated neutral effluents using modified materials. J. Environ. Manage..

[cit4] Sharafi A., Ardejani F. D., Rezaei B., Sargheini J. (2018). Environmental geochemistry of near-neutral waters and mineralogy of zinc and lead at the Angouran non-sulphide zinc mine, NW Iran. J. Geochem. Explor..

[cit5] Derkowska K., Kierczak J., Potysz A., Pietranik A., Pędziwiatr A., Ettler V., Mihaljevič M. (2023). Combined approach for assessing metal(loid)s leaching, mobility and accumulation in a specific near-neutral (pH) environment of a former Cu-smelting area in the Old Copper Basin, Poland. Appl. Geochem..

[cit6] Lizárraga-Mendiola L., González-Sandoval M. R., Durán-Domínguez M. C., Márquez-Herrera C. (2009). Geochemical behavior of heavy metals in a Zn–Pb–Cu mining area in the State of Mexico (central Mexico). Environ. Monit. Assess..

[cit7] Gondwal M. , Kishore N., Soni R., Verma R. K. and Gautam B. P. S., The Chemical Composition of the Water in the Rivers, Lakes, and Wetlands of Uttarakhand, in Current Status of Fresh Water Microbiology, ed. R. Soni, D. C. Suyal, L. Morales-Oyervides and J. Sungh Chauhan, Springer Nature Singapore, 2023, pp. 29–47

[cit8] Hogstrand C. and Wood C., The physiology and toxicology of zinc in fish, in Seminar series-society for experimental biology, Cambridge University Press, 1996, vol. 57, pp. 61–84

[cit9] Oyaro N. , Ogendi J., Murago E. N. and Gitonga E., The contents of Pb, Cu, Zn and Cd in meat in nairobi, Kenya, 2007

[cit10] Zwain H. M., Vakili M., Dahlan I. (2014). Waste Material Adsorbents for Zinc Removal from Wastewater: A Comprehensive Review. Int. J. Chem. Eng..

[cit11] Environment Agency Wales , Metal Mine Strategy for Wales, 2002

[cit12] Natural Resources Wales , Abandoned Mine Case Study: Cwmystwyth Lead Mine, 2016

[cit13] The Coal Authority and Natural Resources Wales, The Metal Mine Programme, 2024

[cit14] Naidu G., Ryu S., Thiruvenkatachari R., Choi Y., Jeong S., Vigneswaran S. (2019). A critical review on remediation, reuse, and resource recovery from acid mine drainage. Environ. Pollut..

[cit15] Howard A. J., Kincey M., Carey C. (2015). Preserving the Legacy of Historic Metal-Mining Industries in Light of the Water Framework Directive and Future Environmental Change in Mainland Britain: Challenges for the Heritage Community. Hist. Environ.: Policy Pract..

[cit16] Kincey M., Gerrard C., Warburton J. (2022). Metals, mines and moorland: the changing lead mining landscapes of the North Pennines, UK, 1700-1948. Post-Mediev. Archaeol..

[cit17] Johnston D. , A metal mines strategy for Wales, 2004

[cit18] Dunham K. , Geology of the Northern Pennine Orefield Volume I—Tyne to Stainmore Economic memoir covering the areas of 1: 50 000 and one-inch geological sheets 19 and 25, and parts of 13, 24, 26, 31, 32 (England and Wales), 1948

[cit19] Nuttall C. A., Younger P. L. (2000). Zinc removal from hard, circum-neutral mine waters using a novel closed-bed limestone reactor. Water Res..

[cit20] Nuttall C. A. and Younger P. L., Assessment and experimental passive treatment of zinc-rich net alkaline minewaters, Nent Valley, UK, in Proceedings, 7th international mine water association congress, Ustron, Poland, 2000, pp. 456-463

[cit21] Nuttall C. A., Younger P. L. Y. (1999). Reconnaissance hydrogeochemical evaluation of an abandoned Pb – Zn orefield, Nent Valley, Cumbria, UK. Proc. Yorks. Geol. Soc..

[cit22] Brown M. , Barley B. and Wood H., Minewater treatment, IWA publishing, 2002

[cit23] Wolkersdorfer C. , Active Treatment Methods for Mine Water, in Mine Water Treatment – Active and Passive Methods, Springer Berlin Heidelberg, 2022, pp. 95–149

[cit24] Younger P. L. , Banwart S. A. and Hedin R. S., Passive Treatment of Polluted Mine Waters, in Mine Water: Hydrology, Pollution, Remediation, Springer Netherlands, 2002, pp. 311–396

[cit25] Salud O., Francisco M., Carlos R. C., José Miguel N., Rafael P.-L., Carlos A. (2021). Eco-sustainable passive treatment for mine waters: Full-scale and long-term demonstration. J. Environ. Manage..

[cit26] Johnson D. B., Kevin B. H. (2005). Acid mine drainage remediation options: a review. Sci. Total Environ..

[cit27] Ata A., Soner K. (2006). Acid Mine Drainage (AMD): causes, treatment and case studies. J. Cleaner Prod..

[cit28] Rashid R., Shafiq I., Akhter P., Iqbal M. J., Hussain M. (2021). A state-of-the-art review on wastewater treatment techniques: the effectiveness of adsorption method. Environ. Sci. Pollut. Res..

[cit29] Iakovleva E., Sillanpää M. (2013). The use of low-cost adsorbents for wastewater purification in mining industries. Environ. Sci. Pollut. Res..

[cit30] Kebede K. K., Bhekie B. M., Titus A. M. M. (2017). Magnetite and cobalt ferrite nanoparticles used as seeds for acid mine drainage treatment. J. Hazard. Mater..

[cit31] Tang S. C. N., Lo I. M. C. (2013). Magnetic nanoparticles: Essential factors for sustainable environmental applications. Water Res..

[cit32] Klimkova S., Cernik M., Lacinova L., Filip J., Jancik D., Zboril R. (2011). Zero-valent iron nanoparticles in treatment of acid mine water from in situ uranium leaching. Chemosphere.

[cit33] Liu J.-f., Zhao Z.-s., Jiang G.-b. (2008). Coating Fe3O4 Magnetic Nanoparticles with Humic Acid for High Efficient Removal of Heavy Metals in Water. Environ. Sci. Technol..

[cit34] Gilbert C., Ayanda O. S., Fatoba O. O., Madzivire G., Petrik L. F. (2019). A Novel Method of Using Iron Nanoparticles from Coal Fly Ash or Ferric Chloride for Acid Mine Drainage Remediation. Mine Water Environ..

[cit35] Giménez J., Martínez M., de Pablo J., Rovira M., Duro L. (2007). Arsenic sorption onto natural hematite, magnetite, and goethite. J. Hazard. Mater..

[cit36] Hu J., Lo I. M. C., Chen G. (2004). Removal of Cr(VI) by magnetite. Water Sci. Technol..

[cit37] Bayer T., Wei R., Kappler A., Byrne J. M. (2023). Cu(II) and Cd(II) Removal Efficiency of Microbially Redox-Activated Magnetite Nanoparticles. ACS Earth Space Chem..

[cit38] da Silva Medeiros D. C. C., Usman M., Chelme-Ayala P., El-Din M. G. (2025). Biochar-enhanced removal of naphthenic acids from oil sands process water: Influence of feedstock and chemical activation. Energy Environ. Sustain..

[cit39] Dhruv M., Siddharth M., Singh S. K. (2015). Magnetic adsorbents for the treatment of water/wastewater—A review. J. Water Process Eng..

[cit40] Almomani F., Bhosale R., Khraisheh M., Kumar A., Almomani T. (2020). Heavy metal ions removal from industrial wastewater using magnetic nanoparticles (MNP). Appl. Surf. Sci..

[cit41] Giménez J., Martínez M., de Pablo J., Rovira M., Duro L. (2007). Arsenic sorption onto natural hematite, magnetite, and goethite. J. Hazard. Mater..

[cit42] Ozmen M., Can K., Arslan G., Tor A., Cengeloglu Y., Ersoz M. (2010). Adsorption of Cu(II) from aqueous solution by using modified Fe3O4 magnetic nanoparticles. Desalination.

[cit43] O'Neill K. E. B., Biswakarma J., Crane R., Byrne J. M. (2025). Recovery of Co (II), Ni (II) and Zn (II) using magnetic nanoparticles (MNPs) at circumneutral pH. Environ. Sci.: Nano.

[cit44] Nordstrom D. K. (2011). Hydrogeochemical processes governing the origin, transport and fate of major and trace elements from mine wastes and mineralized rock to surface waters. Appl. Geochem..

[cit45] Dean M. T. , Browne M. A. E., Waters C. N. and Powell J. H., A lithostratigraphical framework for the Carboniferous successions of northern Great Britain (onshore), RR/10/007, 2011

[cit46] Warrender R., Pearce N. J. G., Perkins W. T., Florence K. M., Brown A. R., Sapsford D. J., Bowell R. J., Dey M. (2011). Field Trials of Low-cost Reactive Media for the Passive Treatment of Circum-neutral Metal Mine Drainage in Mid-Wales, UK. Mine Water Environ..

[cit47] Parker A. J., Milan D. J., McEwen L. J. (2022). Correlating floodplain geochemical profiles with archival historical mining records to establish depositional chronologies of river sediment. Catena.

[cit48] U. S. G. S. , Surface-Water-Quality Data to Support Implementation of Revised Freshwater Aluminum Water-Quality Criteria in Massachusetts, 2018–19, 2023

[cit49] Bevins R. E. , Young B., Mason J. S., Manning D. A. C. and Symes R. F. Mineralization of England and Wales, ed. L. P. Thomas and E. L. Durham, Geological Conservation Review Series, 2010, vol. 36

[cit50] Clarke S. M. , The geology of NY74SE, Nenthead, Cumbria, OR/07/033, 2007

[cit51] Smith S. , Lead and zinc ores of Northumberland and Alston Moor, 1923

[cit52] Maithani D. , Dasila H., Saxena R., Tiwari A., Bhatt D., Rawat K. and Chandra Suyal D., Heavy Metal Pollution in Water: Cause and Remediation Strategies, in Current Status of Fresh Water Microbiology, ed. R. Soni, D. Chandra Suyal, L. Morales-Oyervides and J. Sungh Chauhan, Springer Singapore, 1st edn, 2023

[cit53] Butler B. A. (2009). Effect of pH, ionic strength, dissolved organic carbon, time, and particle size on metals release from mine drainage impacted streambed sediments. Water Res..

[cit54] UKTAG , River Basin Management (2015-21): Updated Recommendations on Environmental Standards, 2013

[cit55] Peng W., Jia L., Pei A., Zheng Y., Yang X., Shengyan P. (2023). Enhanced delivery of remedial reagents in low-permeability aquifers through coupling with groundwater circulation well. J. Hydrol..

[cit56] Adeli M., Yamini Y., Faraji M. (2017). Removal of copper, nickel and zinc by sodium dodecyl sulphate coated magnetite nanoparticles from water and wastewater samples. Arabian J. Chem..

[cit57] Andelescu A., Nistor M. A., Muntean S. G., Rădulescu-Grad M. E. (2018). Adsorption studies on copper, cadmium, and zinc ion removal from aqueous solution using magnetite/carbon nanocomposites. Sep. Sci. Technol..

[cit58] Mohammed A. A., Brouers F., Isra'a Sadi S., Al-Musawi T. J. (2018). Role of Fe3O4 magnetite nanoparticles used to coat bentonite in zinc(II) ions sequestration. Environ. Nanotechnol., Monit. Manage..

[cit59] Almeida-Naranjo C. E., Morillo B., Aldás M. B., Garcés N., Debut A., Guerrero V. H. (2023). Zinc removal from synthetic waters using magnetite/graphene oxide composites. Remediation.

[cit60] Abdus-Salam N., Adekola S. (2018). Adsorption studies of zinc (II) on magnetite, baobab (Adansonia digitata) and magnetite–baobab composite. Appl. Water Sci..

[cit61] Lu C., Chiu H. (2006). Adsorption of zinc(II) from water with purified carbon nanotubes. Chem. Eng. Sci..

[cit62] Cornell R. M. and Schwertmann U., The iron oxides: structure, properties, reactions, occurrences, and uses, Wiley-vch Weinheim, 2003

[cit63] Dzombak D. A. and Morel F. M., Surface complexation modeling: hydrous ferric oxide, John Wiley & Sons, 1991

[cit64] Trivedi P., Axe L., Tyson T. A. (2001). An Analysis of Zinc Sorption to Amorphous versus Crystalline Iron Oxides Using XAS. J. Colloid Interface Sci..

[cit65] Whitaker A. H., Duckworth O. W. (2018). Cu, Pb, and Zn sorption to biogenic iron (oxyhydr) oxides formed in circumneutral environments. Soil Syst..

[cit66] Rong Z., Hang L., Xiaosi S., Xinyue Y., Yun T., Weihong D., Yuyu W., Tiejun S., Xiaofang S. (2025). Freeze–thaw–induced regulation of petroleum hydrocarbon adsorption in cold-region soils: Role of organic matter dynamics. Water Res..

[cit67] Kuichang Z., Kunpeng W., Ryan M. D., Qiyi F., Eva M. D., Xiaochuan H., Ruikun X., Ibrahim A. S., Ze H., Yuren F. (2021). *et al.*, Selective membranes in water and wastewater treatment: Role of advanced materials. Mater. Today.

[cit68] Yang Z., Zhou Z.-w., Guo H., Yao Z., Ma X.-h., Song X., Feng S.-P., Tang C. Y. (2018). Tannic Acid/Fe3+ Nanoscaffold for Interfacial Polymerization: Toward Enhanced Nanofiltration Performance. Environ. Sci. Technol..

