# Supplementary material for: The removal of Zn from complex circumneutral pH mine waters using magnetic nanoparticles (MNPs)
Source: Environ Sci Nano. 2026 Jan 9;13(1):641–53. doi: 10.1039/d5en01049g (PMC12784243; doi:10.1039/d5en01049g)
Supplement: EN-013-D5EN01049G-s001 [file EN-013-D5EN01049G-s001.pdf]

Supplementary Information (SI) for Environmental Science: Nano.
This journal is © The Royal Society of Chemistry 2026

Supplementary information

The removal of Zn from complex circumneutral pH mine waters using magnetic nanoparticles (MNPs)

Katie E.B. O'Neill[1*], Jagannath Biswakarma[1] , Rich Crane[2], James M. Byrne[1*]

[1] School of Earth Sciences, University of Bristol, Bristol, BS8 1TH, United Kingdom

[2]Camborne School of Mines, University of Exeter, Penryn, TR10 9EZ, United Kingdom

[*]Corresponding authors: katie.oneill@bristol.ac.uk; james.byrne@bristol.ac.uk

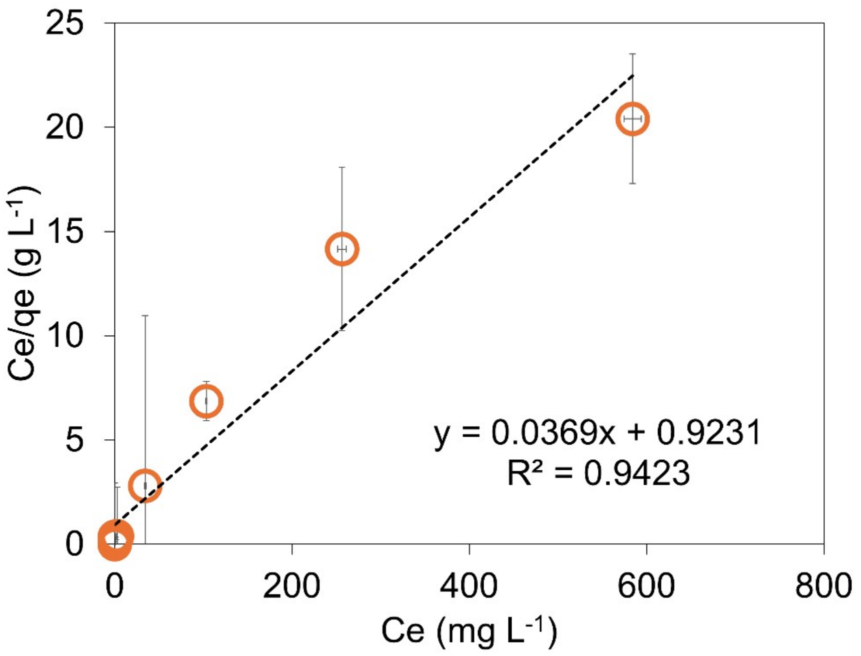

Fig. S1: Linearised Langmuir isotherm which displays the final equilibrium concentration of Zn by the amount of Zn sorbed (C_e/q_e, g L^{-1}) versus Zn final equilibrium concentration (C_e, mg L^{-1}) for 5 g L^{-1}. Experimental data from the previous study by O'Neill et al, 2025[42] and environmental samples from this study are combined and plotted together. The dashed line represents the linear regression fit, with the equation of the line displayed. Error bars represent standard deviations from triplicate measurements.

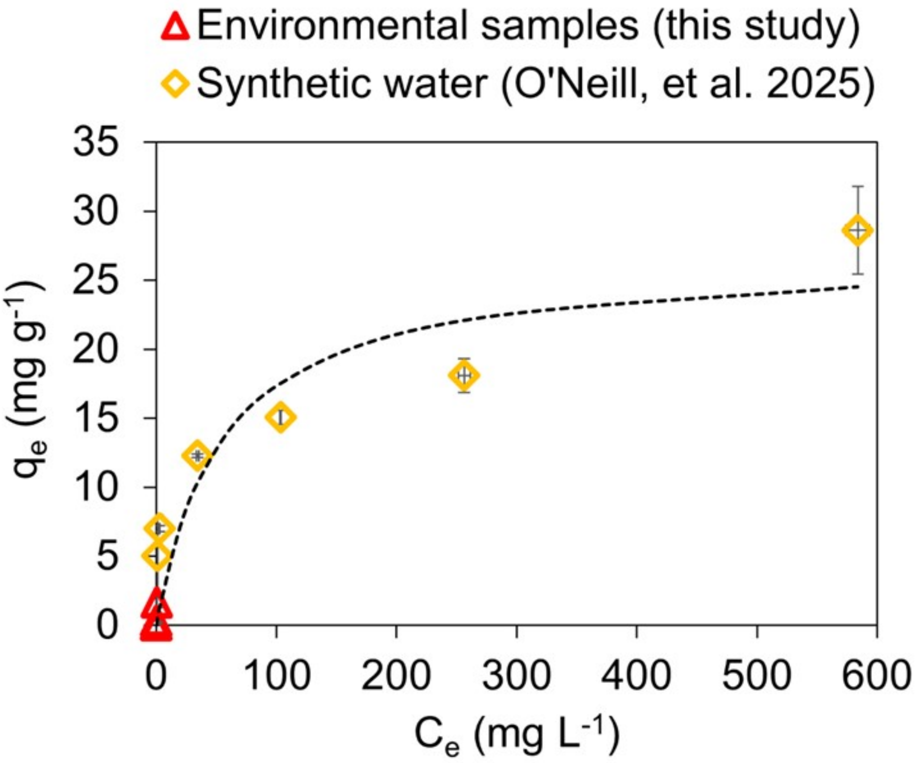

Fig. S2: Sorption of Zn on MNPs (5 g L^{-1}) was studied under anoxic conditions using both synthetic water (yellow diamonds) and natural mine water samples (red triangles). Black dashed line represents the model fit, determined by Langmuir (non-linear equation). Error bars represent standard deviations from triplicate measurements.

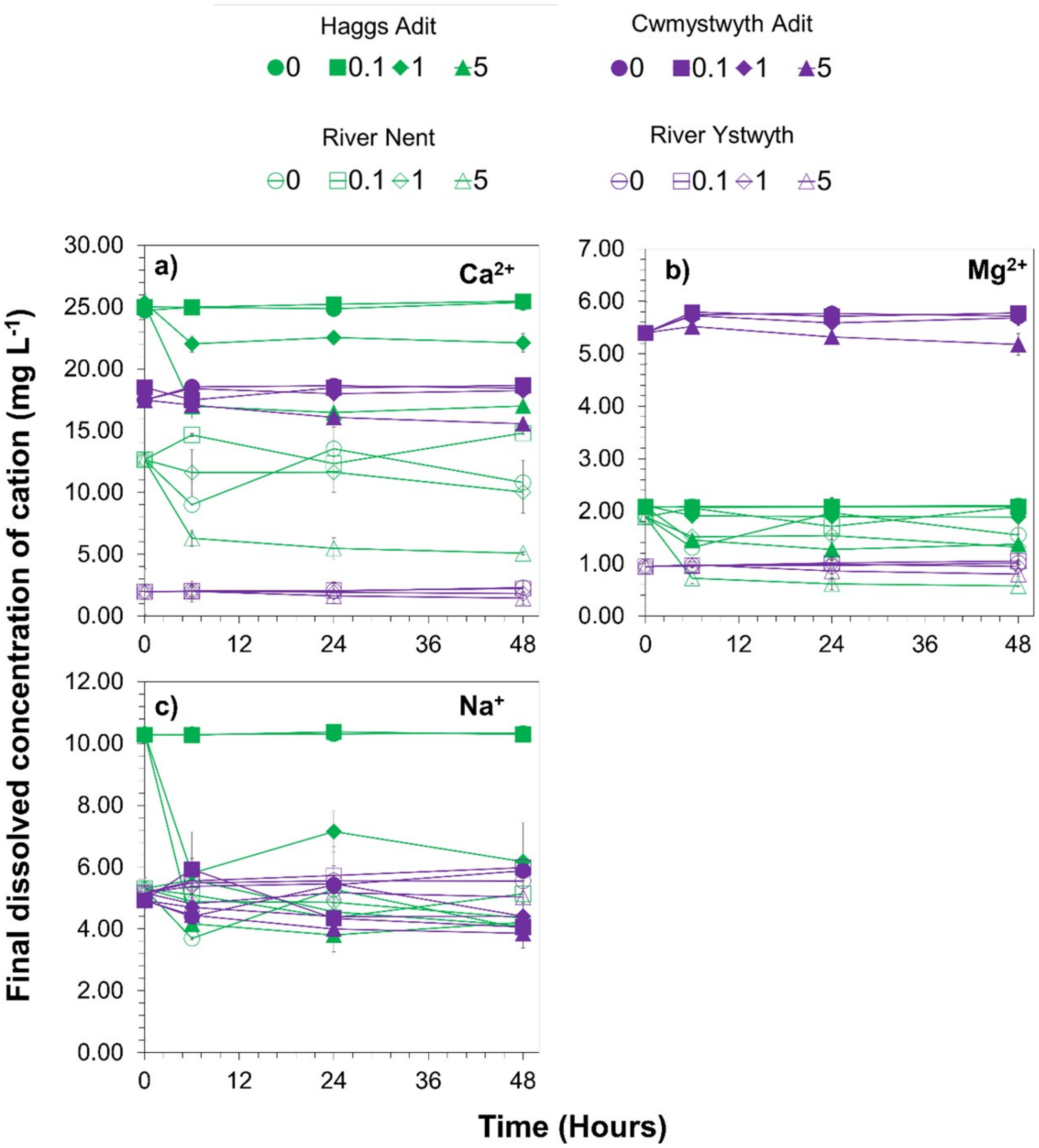

Fig. S3: Final dissolved concentrations of cations (Ca^{2+}, Mg^{2+} and Na^{+}) in water samples from Haggs adit and the River Nent (green) and Cwmystwyth adit and the River Ystwyth (purple) after exposure to varying MNP concentrations (0, 0.1, 1 and 5 g L^{-1}) under anoxic conditions over 48 hours. Final aqueous concentrations are shown for a) Ca^{2+}, b) Mg^{2+} and c) Na^{+}. Symbols indicate MNP concentrations: circles (0 g L^{-1}), squares (0.1 g L^{-1}), diamonds (1 g L^{-1}) and triangles (5 g L^{-1}). Open symbols represent mine adits, while closed symbols represent rivers. Line and symbol colours correspond to site locations: green for Haggs adit and the River Nent, and purple for Cwmystwyth adit and the River Ystwyth in purple. Error bars represent standard deviations from triplicate measurements.

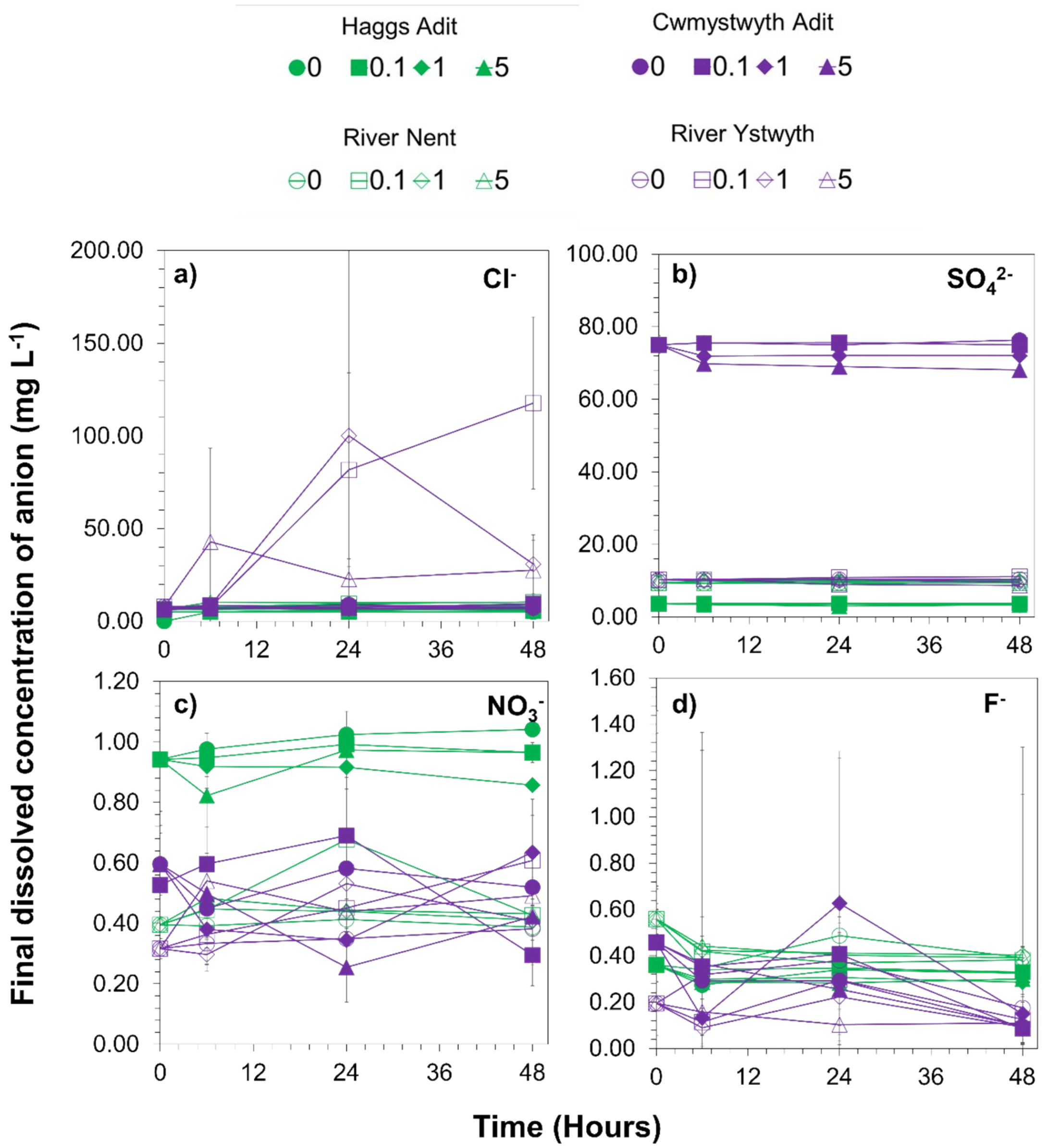

Fig. S4: Final dissolved concentrations of anions (Cl^-, SO_4^{2-}, NO_3^- and F^-) in water samples from Haggs adit and the River Nent (green) and Cwmystwyth adit and the River Ystwyth (purple) after exposure to varying MNP concentrations (0, 0.1, 1 and 5 g L^{-1}) under anoxic conditions over 48 hours. Final aqueous concentrations are shown for a) Cl^-, b) SO_4^{2-}, c) NO_3^- and d) F^-. Symbols indicate MNP concentrations: circles (0 g L^{-1}), squares (0.1 g L^{-1}), diamonds (1 g L^{-1}) and triangles (5 g L^{-1}). Open symbols represent mine adits, while closed symbols represent rivers. Line and symbol colours correspond to site locations: green for Haggs adit and the River Nent, and purple for Cwmystwyth adit and the River Ystwyth in purple. Error bars represent standard deviations from triplicate measurements.

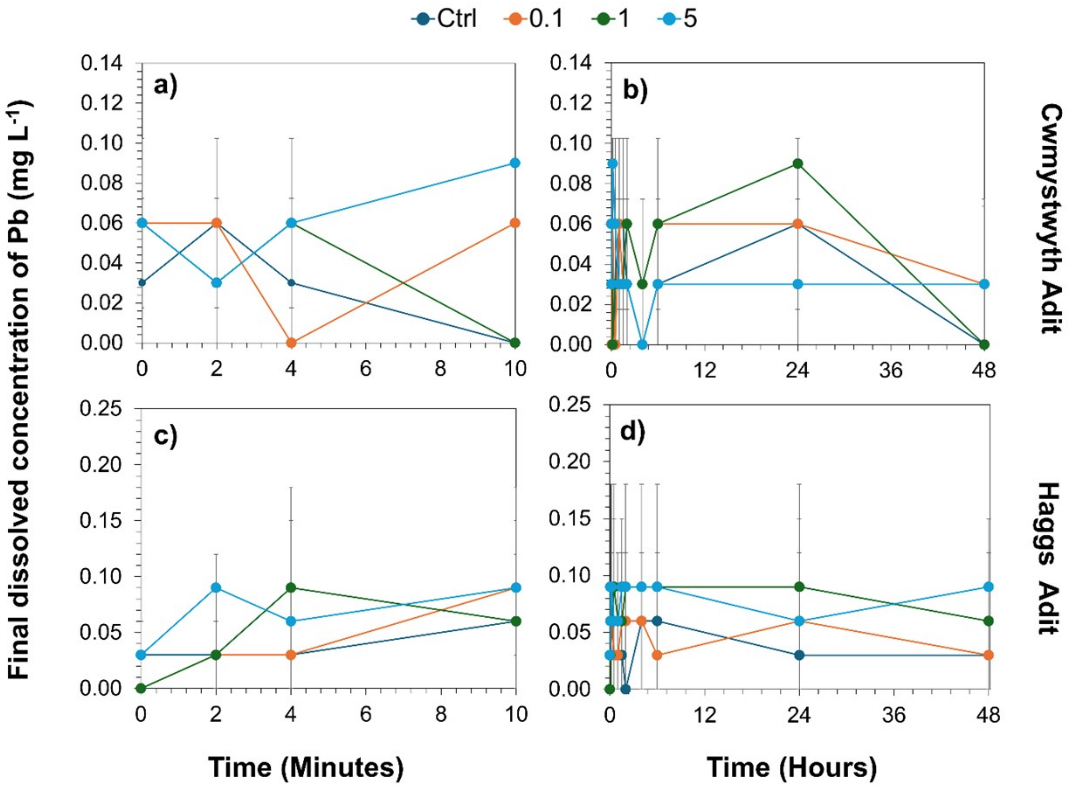

Fig. S5: Final dissolved concentrations of Pb in in water samples from the mine adits. after exposure to varying MNP concentrations (0, 0.1, 1 and 5 g L^{-1}) under anoxic conditions over 48 hours. Final aqueous concentrations are shown for Cwmystwyth adit a) first 10 minutes and b) full 48 hours, and for Haggs adit in c) first 10 minutes and d) full 48 hours. Colours indicate the concentrations of MNPs with 0 g L^{-1} in dark blue, 0.1 g L^{-1} in orange, 1 g L^{-1} in green and 5 g L^{-1} in light blue. Error bars represent standard deviations from triplicate measurements.
